# Chronic Nonbacterial Osteomyelitis in Children

**DOI:** 10.3390/children8070551

**Published:** 2021-06-25

**Authors:** Aikaterini Koryllou, Manel Mejbri, Katerina Theodoropoulou, Michael Hofer, Raffaella Carlomagno

**Affiliations:** 1 Pediatric Immuno-Rheumatology of Western Switzerland, CHUV, University of Lausanne, 1011 Lausanne, Switzerland; manel.mejbri@chuv.ch (M.M.); aikaterini.theodoropoulou@chuv.ch (K.T.); Michael.Hofer@chuv.ch (M.H.); raffaella.carlomagno@chuv.ch (R.C.); 2 Department of Biochemistry, University of Lausanne, 1011 Lausanne, Switzerland

**Keywords:** chronic nonbacterial osteomyelitis (CNO), chronic recurrent multifocal osteomyelitis (CRMO), chronic nonbacterial osteitis, SAPHO, DIRA, PAPA syndrome, Majeed syndrome, monogenic, auto-inflammatory bone disease

## Abstract

Chronic nonbacterial osteomyelitis (CNO) is an auto-inflammatory bone disorder with a wide spectrum of clinical manifestations, from unifocal to multifocal lesions. When it manifests with multifocal lesions, it is also referred to as chronic recurrent multifocal osteomyelitis (CRMO). CNO/CRMO can affect all age groups, with the pediatric population being the most common. Patients may present with systemic inflammation, but there is no pathognomonic laboratory finding. Magnetic resonance imaging (MRI) is the gold standard radiological tool for diagnosis. In the absence of validated diagnostic criteria, CNO/CRMO remains an exclusion diagnosis. Bone biopsy does not show a specific disease pattern, but it may be necessary in unifocal or atypical cases to differentiate it from malignancy or infection. First-line treatments are non-steroidal anti-inflammatory drugs (NSAIDs), while bisphosphonates or TNF-α blockers can be used in refractory cases. The disease course is unpredictable, and uncontrolled lesions can complicate with bone fractures and deformations, underlying the importance of long-term follow-up in these patients.

## 1. Introduction

Chronic nonbacterial osteomyelitis (CNO) is an auto-inflammatory bone disorder, first described in 1972 by Giedion et al. [1] as a subacute and chronic symmetrical osteomyelitis. CNO is an umbrella term that includes conditions manifesting with a wide range of clinical manifestations, from single to multiple bone lesions [2]. Several other definitions have been used over time to refer to this heterogeneous disorder, such as chronic recurrent multifocal osteomyelitis (CRMO), synovitis acne pustulosis hyperostosis osteomyelitis (SAPHO) syndrome, and nonbacterial osteomyelitis [3]. 

As CNO is a rare disease, and presents with a wide spectrum of manifestations, the diagnosis can be challenging. In the following sections, clinical presentation, laboratory and radiological investigations, pathophysiology, and treatment options will be discussed.

## 2. Incidence and Demographics

The incidence of CNO varies across geographic areas, and this is thought to be due to increased awareness of the disease in the regions with higher incidence, rather than ethnic or environmental factors [4]. The median age at diagnosis in pediatric patients is 10 years, with most cases reported between 8 and 13 years [4,5,6,7,8]. In European cohorts, females seem more likely to be affected, with a female-to-male ratio of 2:1 [5,6,7,8,9,10], while in Latin American and Indian series a male prevalence is reported [11,12]. Diagnostic delay is common in pediatric CNO, with a mean interval of 12 months between symptom onset and diagnosis in most pediatric studies [4,6,8,13], possibly related to the rareness of the disease, the discrete findings on examination, and the absence of pathognomonic laboratory studies.

## 3. Clinical Presentation 

CNO usually presents with insidious recurrent bone pain in one or multiples sites [5,6,14]. Pain intensity can be variable, and mostly reported in the morning and during the night, causing sleep disturbance [6,15]. This can lead to a mistaken diagnosis of growing pain and consequently delay the diagnosis of CNO [16]. Pain can be associated with local swelling [11,17] and limb impairment [4,11]. Inflammation may occur at any site of the skeleton, but the metaphysis of long bones is the area most frequently involved in children, as it is reported in 33–66% of cases [4,6,8,17]. Osteomyelitis of the sternum, clavicle, or jaw is suggestive of CNO [6], whereas the skull is almost never affected [18,19]. Spinal involvement is seen in up to a third of cases (20–46%) [10,20] and it is an important prognostic factor, since it can be complicated by vertebral fracture [20]. When multifocal, lesions are asymmetric in approximately 60% of cases [6,15]. Involvement of the clavicle remains unifocal throughout the disease course in about half of the patients in Bhat et al.’s cohort [10]. Other clinical features can be associated with the osteomyelitis in CNO patients; arthritis develops in approximately 40% of the cases, either at onset or later [11,15,17]. Systemic features such as fever or fatigue are reported in 17–20% of patients [3,15]. 

CNO is associated with other chronic inflammatory conditions in up to 20% of cases, including juvenile idiopathic arthritis, spondylarthritis, inflammatory bowel disease, and dermatological manifestations (psoriasis, pustulosis palmaris et plantaris, pyoderma gangrenosum, severe acne, Sweet syndrome) [6,8].

## 4. Laboratory Findings

The percentage of patients presenting with systemic inflammation at CNO onset varies significantly. In particular, the proportion of increased inflammatory markers reported, such as C-reactive protein (CRP) and erythrocyte sedimentation rate (ESR), ranges between 19 and 90% in different studies [4,6,7,8,9,10,14,15,17,19,20,21,22,23]. It is interesting to note that ESR and CRP levels do not seem to be predictive of a particular course of the disease [4,5]. Beck et al. show a correlation between the ESR and the number of radiological lesions, but not with the number of symptomatic sites, suggesting that asymptomatic lesions may be a contributing factor to systemic inflammation [5]. In patients with CNO/CRMO, leukocytosis has been described in 14–20% of cases [17]. The elevation of inflammatory markers in CNO/CRMO is usually moderate, and it resolves during inactive periods [5]. As per other inflammatory biomarkers, ferritin is commonly within normal ranges, even though its levels were reported as significantly higher in the initial examination versus follow-up of CNO/CRMO patients by Beck et al. [5]. No relevant elevation of IgD, IgG, IgA, or IgM has been shown [8].

HLA-B27 positivity is reported in a small percentage of CNO/CRMO patients: 7.5% in the large pediatric registry of Eurofever [8], 7% in a large national French cohort of 178 patients [6], and 6% in a Chilean series [11]. This observation, together with the axial involvement in some patients, led several authors to suspect an association between spondylarthritis and CRMO [24,25]. Nevertheless, in all studies, presence of HLA-B27 does not correlate with the presence of arthritis or axial skeleton involvement [11], nor with a more severe disease course [5]. 

Recently, some authors have discussed the role of serum cytokines and chemokines, such as S100A8/A9, in differentiating CNO from other conditions such as leukemia, infections, IBD, and healthy control [26,27]. More studies are required to evaluate the use of these biomarkers in the process of diagnosis, treatment strategy, and follow-up in CNO patients.

## 5. Radiological Assessment

Radiological assessment is particularly important in diagnostic evaluation since CNO/CRMO is a diagnosis of exclusion, and its early symptoms can overlap with other conditions (e.g., bone tumors). Different radiological tools can be used for the diagnosis, but whole-body magnetic resonance imaging (MRI) remains the gold standard [4,8,13]. 

MRI offers a more sensitive method in detecting inflamed sites, with the advantages of avoiding radiation exposure [8,13] and evaluating both disease activity and possible skeletal complications. Furthermore, lesions can be asymptomatic in 47–64% of patients [4,10], in particular in the early stages of the disease. This underlines the importance of imaging for an early detection of silent lesions and their complications, especially spinal involvement (fracture, vertebra plana) [28]. The most frequent radiologic findings described on MRI are bone marrow edema [13] and osteolytic lesions [4,9,17,28]. Long bones metaphyses, especially the distal tibial metaphysis, are the most involved sites in all studies [2,4,10,19]. 

Standardizing imaging characteristics in CNO patients is important in order to develop a grading system and facilitate disease assessment. Different tools have been described in the past years (RINBO, CROMRIS) using mainly the following characteristics: bone edema and soft tissue inflammation extent, evaluation of periosteal reaction, hyperostosis, growth plate damage, and vertebral compression [29,30,31]. None of these grading systems have been validated.

X-ray is often the first exploratory tool used in the process of bone pain investigation in children. X-ray abnormalities, such as modifications in bone metaphyses and osteolytic lesions, are described in the literature in more than 50% of CNO/CRMO patients [4,10,17]. However, these are not specific changes [9] and are usually not detected in the early stages of the disease [14]. Therefore, X-ray seems helpful as a first-line tool but MRI is preferred for its sensitivity and lack of radiation [17].

A computer tomography (CT) scan is more sensitive than X-ray. However, taking into account its considerable radiation exposure, it is mostly considered an alternative diagnostic tool in case MRI is not available [13]. Similarly, scintigraphy can also be used as a second-choice imaging in case of non-availability of MRI.

## 6. Bone Biopsy

Surgical bone biopsy may be necessary to exclude other diseases such as malignancy or infection, in particular in unifocal and/or atypical locations, in cases of earlier onset (less than 2 years) or atypical evolution under treatment [13,14,32]. When biopsy is indicated, common CNO histological findings are not pathognomonic, and include edema, hyperemia, infiltration of lymphocytes and plasma cells compatible with non-specific chronic inflammation, marrow fibrosis, and osteonecrosis [11,17,32].

## 7. Diagnosis

Different teams have proposed diagnostic criteria for CNO [32,33], but these have not been validated in prospective studies.

To date, CNO/CRMO remains a diagnosis of exclusion. The evidence of multifocal involvement can contribute in differentiating CNO/CRMO from other diseases such as bacterial osteomyelitis. However, in unifocal or atypical cases, a bone biopsy may be required [32].

**Figure 1:**
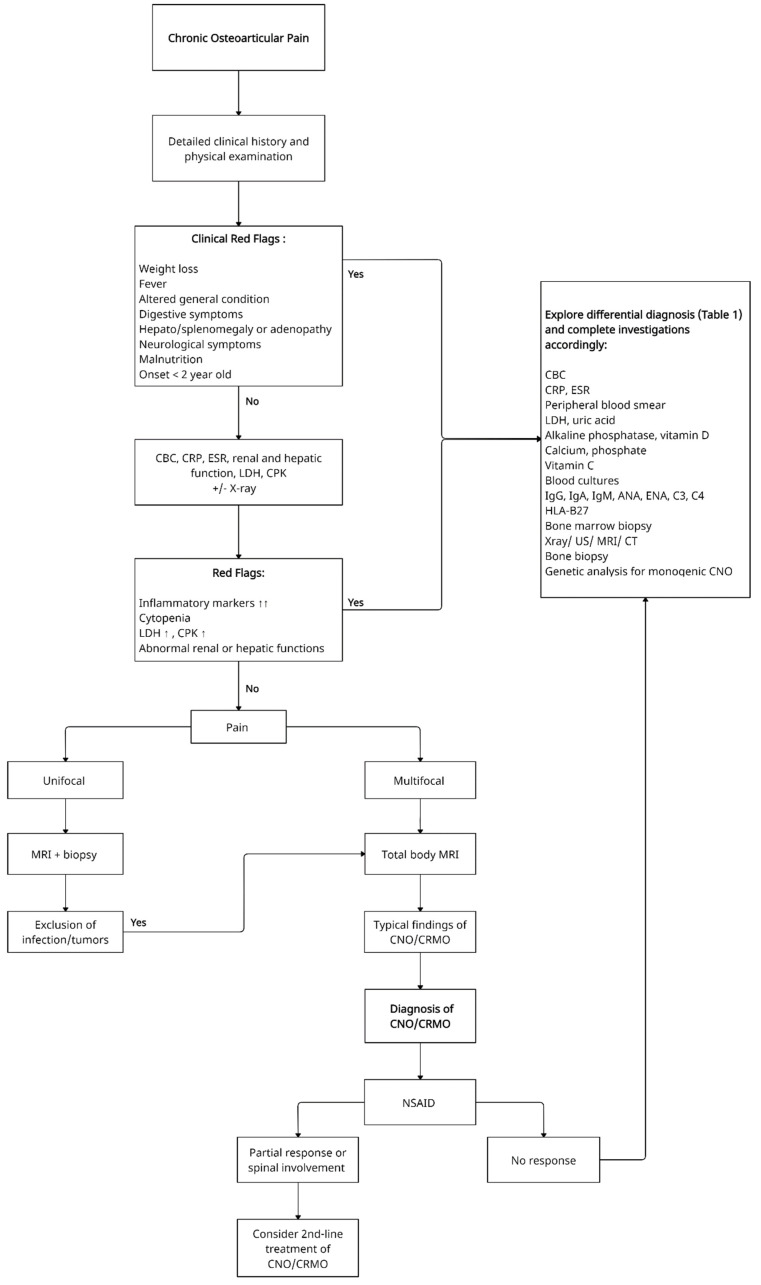
Proposed schematic decisional algorithm for patients with unifocal/multifocal osteoarticular pain. CBC—Complete blood count, CRP—C-reactive protein, ESR—Erythrocyte sedimentation rate, LDH—Lactate dehydrogenase, CPK—Creatine phosphokinase, ANA—Antinuclear antibodies, ENA—Extractable nuclear antigen antibodies panel, US—Ultrasound, MRI—Magnetic resonance imaging, CT—Computed tomography, CNO—Chronic non-bacterial osteomyelitis, CRMO—Chronic recurrent multifocal osteomyelitis, NSAID- Non-steroidal anti-inflammatory drug.

A proposed schematic decisional algorithm for patients with unifocal/multifocal osteoarticular pain is presented in Figure 1. The most common differential diagnoses that have to be considered are presented in Table 1.

## 8. Pathophysiological Mechanism of CNO

Osteolytic lesions and bone resorption may be the result of the serum pro-inflammatory cytokines pattern found in CNO patients. These cytokines may activate osteoclasts via receptor activator of nuclear factor kappa-B (RANK) and RANK ligand signaling. 

Over the past years, Hofmann and his colleagues have illustrated the role of cytokine imbalance between the pro- and anti-inflammatory cytokines in the pathophysiology of CNO. They demonstrated that, when stimulated, peripheral blood monocytes from CNO patients produce lower levels of immune-regulatory cytokines, including IL-10 and IL-19, compared to monocytes from the control group. They also published data showing increased levels of pro-inflammatory cytokines (IL-1, IL-6, and TNFα) and decreased levels of IL-10 and IL-19 in patients’ sera [26,34,35,36,37,38]. Reduced IL-10 transcription due to epigenetic alterations resulting in the ‘closure’ of the IL-10 promotor was also reported by the same research group [34].

Furthermore, Scianaro et al. suggest an abnormal regulation of the IL-1β axis with a potential implication of the NLRP3 inflammasome in CNO pathogenesis [39].

A genetic predisposition for CNO has been widely suggested. Wipff et al. reported a prevalence of inflammatory bone disease among patients’ relatives ranging between 12 and 32% in large cohorts of patients with CNO [6]. The occurrence of multiple affected members and a high incidence of psoriasis, inflammatory bowel disease, and other chronic inflammatory conditions in first-degree relatives underline the genetic burden among these patients [40,41]. 

Hofmann et al. analyzed polymorphisms in the IL-10 proximal promoter region in a cohort of CNO patients, resulting in an enrichment of IL-10 promoter haplotypes encoding for high IL-10 expression, while no single CNO patient was found with homozygous low IL-10 expression haplotypes in this cohort [35].

Cox et al. analyzed the whole exome sequence in a CNO cohort and reported a variant in the filamin-binding domain of the FBLIM1 gene in two unrelated CNO patients from South Asia [42,43]. Both patients carried IL-10 promoter haplotypes encoding for low IL-10 expression. The hypothesis was evoked that the combination of IL-10 promoter haplotypes encoding for low gene expression together with FBLIM1 variants may predispose patients to CNO [37].

Evidence of genetic burden has further been suggested by clinical similarities between the sporadic and the monogenic forms of CNO, detailed below.

## 9. Monogenic Forms of CNO 

### 9.1. Majeed Syndrome

Majeed syndrome is a rare auto-inflammatory genetic disorder first described in 1989. The clinical triad of early onset CNO, microcytic congenital dyserythropoietic anemia, and neutrophilic dermatosis is the hallmark of this disease [44]. However, a considerable phenotypic variability in the clinical presentation has become evident over the last years, with less than 10% of reported cases having all three clinical features [45]. Ferguson et al. report a median age at onset of 12 months, and an average age at onset of 20.4 months. Majeed syndrome is an autosomal recessive disorder due to a loss of function mutation in the LIPIN2 gene. Lipin-2 is one of the LIPIN family proteins (Lipin-1, Lipin-2, Lipin-3) having a central role in lipid metabolism [46]. Ιn 2017, Lorden et al. demonstrated that Lipin-2 has an important role in the activation of the NLRP3 inflammasome and the regulation of IL-1β production in primary human and mouse macrophages, by several mechanisms: it inhibits the activation and the sensitization of the purinergic P2X7 macrophage receptor, inhibits the inflammasome assembly, regulates mitogen-activated protein kinases (MAPK) activation, and controls caspase-1 activation [46]. Given the crucial role of pro-inflammatory cytokine IL-1 in Majeed syndrome, some authors classify it as an NLRP3 inflammasomopathy [45,46,47]. IL-1 blockers have been used in patients with Majeed syndrome, and significant benefit has been noted. Authors report resolution of inflammatory bone disease, normalization of inflammatory biomarkers, and improvement in the anemia [47,48,49,50,51,52].

### 9.2. Interleukin-1 Receptor Antagonist Deficiency (DIRA)

DIRA is a rare auto inflammatory disease first described in 2009. It is characterized by neonatal onset of severe neutrophilic skin pustulosis, sterile multifocal osteomyelitis, periostitis, and elevated inflammatory markers. DIRA is an autosomal recessive disorder due to homozygous mutations in the IL1RN gene encoding for the interleukin-1 receptor antagonist (IL-1Ra) [53]. This results in deficiency in IL-1Ra with continuous activation of the pro-inflammatory cytokines IL-1α and IL-1 β. 

DIRA is a severe, life-threatening, but treatable inflammatory disease [53,54]. IL-1 blockage with anakinra leads to a rapid and durable clinical and biological remission. The use of other IL-1 blockers such as canakinumab and rilonacept has showed similar results. To date, there is no available study that compares the long-term efficacy among the different IL-1 blockers [53,54,55,56,57,58].

Recently, Kuemmerle-Deschner et al. reported the case of an atypical DIRA presentation, CNO-like with a later clinical onset at 1 year of age. This reported case increases the spectrum of DIRA presentation and highlights the importance of considering serum IL-1Ra dosage in patients with early-onset CNO-like bone lesions and biologic inflammation, even without skin manifestations [59].

### 9.3. PAPA Syndrome

PAPA syndrome is a rare auto inflammatory disease usually manifesting early in life with pyogenic arthritis, pyoderma gangrenosum, and cystic acne. It is an autosomal dominant disease caused by a mutation in the proline serine threonine phosphatase-interacting protein 1 (PSTPIP1) gene, leading to overproduction of the pro-inflammatory cytokine IL-1 [60,61]. Sporadic cases of aseptic osteomyelitis with bone lesions similar to those found in CNO patients are reported in some cases of PAPA syndrome [60,61,62,63,64]. However, this is not considered as a genetic cause of CNO by all authors. Glucocorticoids are standard treatment in patients with PAPA syndrome, and good results are also reported with TNF-α and IL-1 blockers [60,62,63,64].

## 10. Treatment

The aims of CNO treatment are pain management, improvement in inflammation, and prevention of complications. Since most of the previously published studies are mainly small retrospective case series, the treatment of CNO remains empirical. 

### 10.1. NSAIDs

Non-steroidal anti-inflammatory drugs (NSAIDs) are frequently suggested as a first-line treatment in CNO patients, especially in children, known to respond better than adults to them [17]. Naproxen is the most prescribed NSAID, usually at a dose of 10 mg/kg (maximum 500 mg) twice daily [13], and is usually well tolerated [5,7]. It is suggested that it should be maintained for at least one month before evaluating the response [19]. Many retrospective studies have shown that NSAIDs produce a clinical improvement and can achieve control of the symptoms or clinical remission in more than 50% of all patients within the first 12 months [6,8,20,22,23,25,65]. Beck et al. confirmed the previously described results in a prospective study, showing significant clinical improvement in the first year of treatment in 43% of the patients, and a decrease in the number of radiological bone lesions in the first three months [5]. Nevertheless, this treatment seems less effective in cases of CNO with spinal involvement. The recent study of Kostik et al., including 91 children (31.9% of whom with spinal lesions), showed a moderate effect of NSAIDs in patients with spinal involvement. NSAIDs therefore seem mainly effective in CNO with peripheral involvement, particularly unifocal forms or clavicle involvement [20]. In cases of persistent bone pain or systemic inflammation despite 3 months of NSAID treatment, a second-line treatment should be considered [66].

### 10.2. Glucocorticoids

Glucocorticoids (GCs) can be used as a short-term treatment when symptoms are not controlled with NSAIDs, once other diagnoses have been ruled out [66]. GCs are not recommended as a long-term treatment due to the well-known side effects.

Second-line treatments include methotrexate, TNF-α blockers, and bisphosphonates.

### 10.3. DMARDs

Disease-modifying antirheumatic agents, such as methotrexate, have been used in CNO for their anti-inflammatory effect. Nevertheless, their success is extremely variable among the different studies: Jansson et al. [15] and Borzutzky et al. [3] reported a clinical remission only in 20% of patients treated with methotrexate; Wipff et al. [6] showed an efficacy of 38%; Kaiser et al. [25] documented a positive outcome in 15% of patients; and Girschick et al. [8] showed a complete remission of symptoms in only 22% of the patients treated with methotrexate.

Better results are described by Gamalero et al. [4], with a response rate to methotrexate reported of 66%, and 83% of patients achieving at least a partial disease remission. Concha et al. [11] describe an improvement in 50% of patients receiving methotrexate, but half of them received steroids at the same time.

### 10.4. Biphosphonates

Bisphosphonates (BPs) have been proved an effective treatment for CNO lesions, as they have been used in these patients for almost 20 years. The most studied one is pamidronate, usually prescribed at a dose of 1 mg/kg/dose (maximum 60 mg/dose) every month, or 1 mg/kg/dose (maximum 60 mg/dose) for 3 consecutive days every 3 months [13]. The main benefits of treatment with BPs are related to their anti-inflammatory and pain-relief effects. Their mechanism in CNO is still not clear but is thought to be related to the decrease in osteoblast and osteocyte apoptosis, the reduction in bone resorption, and the increase in secondary bone mineralization [7,10]. The first retrospective studies concerning BPs’ use in CNO patients showed their efficacy in more than half of the children treated with pamidronate after failing NSAIDs [30,64,65,66,67,68]. More recent retrospective studies confirm the previous results, with remission achieved in 69.4–91% of patients [4,7,8,17,20,65]. A high efficacy of BPs in CNO patients with vertebral involvement has been proved in different older studies [28,69]. More recently, Bhat et al. showed a remarkably high response rate in vertebral lesions after pamidronate treatment, with 82.3% of the lesions resolving completely, suggesting that pamidronate could be proposed as a first-line treatment in cases of spinal and mandibular involvement [7,20]. Kostik et al. [20] report a higher efficacy of bisphosphonates, compared to TNF-α blockers, in CNO patients with spinal involvement. Recently, in the first randomized, double-blinded, placebo-controlled trial in patients with CNO treated with pamidronate, Andersean et al. [70] showed an association between the number of lesions and the clinical/radiological response to treatment, therefore suggesting that patients with multifocal CNO could be more eligible for bisphosphonate treatment. 

### 10.5. Biological Therapy

TNF-α blockers have been used lately for cases of CNO having failed other treatments, and this based on the evidence of increased serum TNF-α concentrations in patients with active disease [14,15]. Studies over the years have evaluated the efficacy of TNF-α blockers in CNO (etanercept, adalimumab, infliximab), mainly after failure of other treatments, and have shown an efficacy ranging between 46 and 89%, shown by clinical remission in 3 months [3,6,15,71]. More recent studies have confirmed these positive observations, reporting an efficacy ranging between 50 and 90.9% of cases [8,10,17,23,65].

During one of the latest international consensus meetings, the efficacy of pamidronate and TNF-α blocker (adalimumab) was estimated to be very similar [72].

As IL-1β seems involved in the pathophysiological mechanism of the disease, interleukin-1 (IL-1) blockers have been studied as a treatment option in CNO. However, the few cases reported show variable responses [2,25,48,71,73]. Nevertheless, as previously mentioned, IL-1 blockers are considered as an effective treatment in monogenic forms of CNO.

The presence of only few prospective studies and the difficulties in assessing disease activity, with a non-uniform definition of treatment efficacy and remission, make it challenging to provide valid therapy and assessment protocols. Recently, consensus treatment plans have been developed by the Childhood Arthritis and Rheumatology Research Alliance (CARRA) group, based on the best available evidence and current treatment practices of North American pediatric rheumatologists for the treatment of pediatric CNO refractory to NSAIDs and/or with active spinal lesions. These consensus plans consist of three possible treatment regimens for patients unresponsive to NSAID: (1) methotrexate or sulfasalazine, (2) TNF-α inhibitors with/without methotrexate, and (3) bisphosphonates; short courses of glucocorticoids and continuation of NSAIDs are permitted in all regimens [13]. This consensus will allow future comparative studies, and help identifying efficacious treatment protocols [7,13,20].

## 11. Physical Activity and Quality of Life

Previous studies on pediatric rheumatic diseases, such as juvenile idiopathic arthritis (JIA), showed that increased levels of activity and exercise in children can restore normal mechanical, physical, and biochemical processes and have a significant effect on reducing symptoms, regardless of the type of exercise [74,75]. Besides these benefits, physical exercise may prevent the loss of bone mineral density associated with chronic inflammation [76]. This highlights the benefit of regular physical activity, eventually with a structured physiotherapy program, in all patients with chronic inflammatory osteoarticular conditions, including CNO.

Nentwich et al. reported the assessment of physical activity, fitness, and health-related quality of life (HRQoL) in 15 patients with CNO, compared to 1:1 matched healthy controls. Their results interestingly show significantly lower scores in self-reported measures of physical activity and HRQoL in CNO patients, even in clinical and/or radiological remission, and this despite similar results in the two groups of the exercise test and accelerometry. The authors suggest that psychological factors may contribute to the level of physical activity in CNO patients, and also underline the need for psychosocial support during treatment [77]. 

A negative impact of CNO on daily life, including family relationships, friendships, and work/school, was also reported by two recent patient surveys, highlighting the need for psychosocial support [78,79].

## 12. Evolution and Clinical Monitoring

The disease course is unpredictable and often marked by acute exacerbations and spontaneous remissions [70]. In the largest pediatric registry, it is described as continuous in 42%, recurrent in 52%, and continuous and recurrent in 5% of patients [8].

Clinical monitoring and assessment of disease activity remain unclear. Different criteria have been reported to define clinical responses to treatments in CNO [5,21]. Most of these criteria include three main components: pain/active lesions, inflammatory markers (ESR and CRP), and imaging findings [5,15]. Considering the delay between the clinical and radiological improvement in lesions, in clinical practice treatment response is mainly monitored by the clinical symptoms and the normalization of ESR and CRP [8,80]. 

The significance of asymptomatic lesions is not yet clear. Most physicians do not rely on asymptomatic lesions for therapeutic decisions unless they concern spinal sites, which could potentially lead to complications such as spinal fracture [66]. 

According to the CARRA consensus it is proposed that follow-up should be pursued at a minimum interval of 3 months for the first year, or more often in cases of suboptimal clinical course. MRI can be particularly useful for assessing follow-up, especially after treatment initiation [32]. MRI is strongly recommended at 6 and 12 months after adjusting therapy, or earlier in cases of persistent activity [13]. When available, whole-body MRI is preferred. Once remission is reached, treatment effect can be assessed with an MRI every 1–2 years [8].

## 13. Prognosis

Long-term prognosis of CNO has been reported to be generally favorable, with remission observed in 40% of patients after 1–5 years of follow-up [3,6,11]. A more recent study comparing the pediatric and adult population, with a median follow-up of 4.8 years, showed a 62.5% remission at last follow-up visit in the pediatric population. Complications were observed in 33.3% of children, and they included vertebral fracture, bone deformity, and chronic pain.

No predictive score of disease severity has been defined. As described by Wipff et al. [6], a diagnostic delay seems to be associated with a worse outcome. 

Disease flares are frequent, and reported in 50–83% of cases [3,11,65]. In a study following the long-term evolution of adult patients with CNO, recurrence is observed even 15 years after disease onset, highlighting the importance of a long-term follow-up and monitoring, and an attentive and throughout transition to adult care [11].

## 14. Conclusions

Chronic nonbacterial osteomyelitis is an inflammatory bone disorder resulting from the imbalance of cytokine secretion from innate immune cells. Clinical manifestations range from asymptomatic/mild symptoms to severe pain, and from a single lesion to multifocal involvement. Whole-body MRI is the gold standard for the diagnosis and follow-up of CNO patients. Even if CNO remains a diagnosis of exclusion, since diagnostic criteria or pathognomonic disease biomarkers are not available, it is important to consider CNO in the differential diagnoses in a child with persistent or intermittent bone and/or joint pain. An early diagnosis is important in order to avoid serious complications such as vertebral fractures and chronic pain. Recently, consensus treatment plans for the treatment of CNO in patients refractory to NSAIDs and/or with active spinal lesions have been developed by CARRA. 

Additional research is needed to investigate the use of biomarkers in diagnostic processes and disease activity, and to optimize treatment and follow-up protocols.

## Figures and Tables

**Table 1:** Differential diagnosis of CNO/CRMO. CNO—Chronic non-bacterial osteomyelitis, CRMO—Chronic recurrent multifocal osteomyelitis.

Common Differential Diagnosis of CNO/CRMO:
**Primary malignant bone diseases:** Ewing sarcoma Osteosarcoma Bone metastases Primary non-Hodgkin lymphoma of bone **Benign bone diseases:** Osteoid osteoma Osteoblastoma Chondroblastoma Cystic bone tumor **Hematological diseases:** Leukemia Lymphoma Langerhans cell histiocytosis of bone Non Langerhans cell histiocytosis **Metabolic diseases:** Hypophosphatasia Vitamin C deficiency **Auto-inflammatory diseases:** Chronic arthritis PSTPIP1-associated auto-inflammatory diseases **Others:** Infectious osteomyelitis Avascular necrosis (osteonecrosis) Amplified musculoskeletal pain syndrome/complex regional pain syndrome Growing pain Cherubism Fibrous dysplasia

## Data Availability

Not applicable.
